# The role of interleukin-18 in the metabolic syndrome

**DOI:** 10.1186/1475-2840-9-11

**Published:** 2010-03-23

**Authors:** Marius Trøseid, Ingebjørg Seljeflot, Harald Arnesen

**Affiliations:** 1 Center for Clinical Heart Research, Department of Cardiology, Oslo University Hospital, Ullevål, Oslo, Norway; 2 Faculty of Medicine, University of Oslo, Oslo, Norway

## Abstract

The metabolic syndrome is thought to be associated with a chronic low-grade inflammation, and a growing body of evidence suggests that interleukin-18 (IL-18) might be closely related to the metabolic syndrome and its consequences. Circulating levels of IL-18 have been reported to be elevated in subjects with the metabolic syndrome, to be closely associated with the components of the syndrome, to predict cardiovascular events and mortality in populations with the metabolic syndrome and to precede the development of type 2 diabetes. IL-18 is found in the unstable atherosclerotic plaque, in adipose tissue and in muscle tissue, and is subject to several regulatory steps including cleavage by caspase-1, inactivation by IL-18 binding protein and the influence of other cytokines in modulating its interaction with the IL-18 receptor. The purpose of this review is to outline the role of IL-18 in the metabolic syndrome, with particular emphasis on cardiovascular risk and the potential effect of life style interventions.

## Introduction

The metabolic syndrome is a cluster of risk factors that identifies a population with increased risk for developing type 2 diabetes mellitus and cardiovascular disease (CVD). The syndrome has received increased attention after practical definitions by the Adult Treatment Panel III and International Diabetes Federation [1,2]. The diagnostic criteria vary slightly, in particular concerning the meausure for central obesity, but in a recent statement it is agreed that the presence of any three of the following five components is diagnostic for the metabolic syndrome: Central obesity, elevated blood pressure, raised fasting glucose, raised levels of trigycerides and low levels of high-density lipoprotein cholesterol [3]. More than 25% of the US population can be classified as having the metabolic syndrome, and the prevalence is increasing with age, affecting >40% of US adults above the age of 60 years [4-6].

The metabolic syndrome is a strong predictor of type 2 diabetes, with an increased incidence rate of 5 to 7-fold [7,8]. Indeed, the increased cardiovascular risk might develop as a continuum in parallel with increasing fasting glucose, from the normal range via impaired fasting glucose to overt diabetes mellitus [9]. The risk of developing CVD is approximately doubled in the metabolic syndrome [10]. In a meta-analysis including 43 cohorts, the relative risk for cardiovascular events and death was 1.78, with the highest risk in women [11].

There is increasing evidence that the metabolic syndrome is associated with a chronic, low-grade inflammation [2]. Several pro-inflammatory cytokines have been shown to be elevated in parallel with an increasing number of components of the syndrome, whereas the anti-inflammatory and adipocyte-specific substance adiponectin is consistently lower [12-15]. Some investigators have discussed that both type 2 diabetes, metabolic syndrome and atherosclerosis are multifactorial conditions which appear to have a common inflammatory basis [16], and it is currently discussed if a measure of inflammation should be included in the definition of the syndrome [2,17]. So far, CRP has been the most likely candidate [17,18].

However, a growing body of evidence suggests that IL-18 is closely associated with the metabolic syndrome and its consequences. The purpose of this review is to outline the role of IL-18 in the metabolic syndrome, with particular emphasis on CVD risk and life style interventions.

### Interleukin-18: a unique proinflammatory cytokine

IL-18 is a member of the IL-1 family of cytokines and was originally described as an interferon gamma (IFN-γ) inducing factor [19]. The cytokine is produced constitutively in many different cell types, including macrophages, endothelial cells, vascular smooth muscle cells, dendritic cells and Kupffer cells [20-22]. IL-18 is also produced in adipocytes [23], but non-adipocyte cells have been identified as the main source of IL-18 in adipose tissue [24]. In contrast to most other cytokines, but in a similar way to IL-1β, IL-18 is expressed as a precursor, pro-IL18, which is inactive until cleaved by the enzyme caspase-1 [25]. Notably, caspase-1 itself exists as an inactive precursor which requires the assembly of multi-unit complexes, known as inflammasomes, to be activated [26,27].

Once secreted, IL-18 is bound and inactivated by IL-18 binding protein, which is enhanced as a negative feedback mechanism in response to increased IL-18 production, ensuring protection from tissue damage due to uncontrolled proinflammatory activity [28,29]. IL-18 binds to its receptor, consisting of an α chain which is responsible for extracellular binding of IL-18, and a β chain which is responsible for intracellular signal transduction [25,30,31]. Although both free and protein-bound IL-18 may bind to the α chain, only the free fraction is able to activate the β chain [28,29].

**Figure 1:**
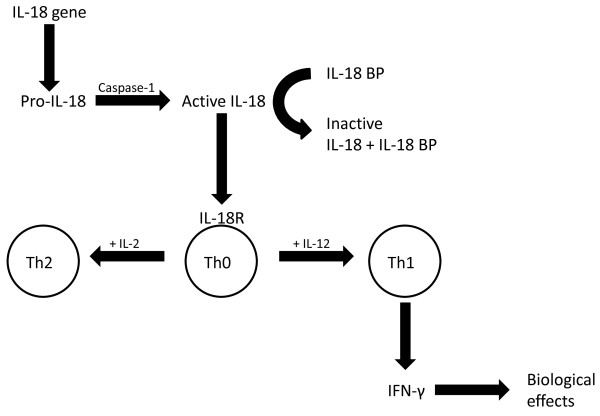
**Regulation and biological effects of interleukin-18**. The cytokine is expressed as a precursor, pro-IL-18, which is inactive until cleaved by caspase-1. Once secreted, IL-18 is bound and inactivated by IL-18 binding protein (IL-18 BP), and only the free fraction can stimulate a signal transduction via the β-chain of the IL-18 receptor (IL-18R). The biological effect is dependent on the cytokine milieu: IL-18 may stimulate a Th2 response in combination with IL-2, and may act synergistically with IL-12 to stimulate a Th1 response with production of IFN-γ, a central feature of the atherosclerotic lesion.

IL-18 is a potent proinflammatory cytokine which enhances T cell and natural killer cell maturation, as well as the production of cytokines, chemokines and cell adhesion molecules [32,33]. Of note, in CD4+ lymphocytes IL-18 can stimulate both type 1 helper T (Th1) and Th2 responses depending on its cytokine milieu: IL-18 may stimulate a Th2 response in combination with IL-2, and may act synergistically with IL-12 to stimulate a Th1 response with production of IFN-γ [25], a central feature in the atherosclerotic lesion (Figure 1). One mechanism underlying this synergistic effect is that IL-12 may induce the α chain of the IL-18 receptor in lymphocytes [34,35], whereas in non-T cells such as macrophages, the IL-18 receptor is constitutively expressed.

### Interleukin-18 in the metabolic syndrome and type 2 diabetes

IL-18 has in several studies been associated with obesity [14,36-38], insulin resistance [39-41], hypertension [42] and dyslipidemia [14,37]. Furthermore, IL-18 has been shown to be elevated in subjects with the metabolic syndrome [43] and to increase in parallell with an increasing number of components of the syndrome [38]. In a large cross sectional study, elevated IL-18 levels were associated with increasing number of components also after adjustment for insulin resistance, obesity, IL-6 and CRP [14]. Polymorphisms in the IL-18 gene have been shown to be associated with circulating IL-18 levels [44]. Interestingly, a recent study showed that one such polymorphism was associated with increased serum levels of IL-18, impaired insulin sensitivity and increased risk of having the metabolic syndrome [45], suggesting that IL-18 might be involved in the pathogenesis of the syndrome.

Circulating levels of IL-18 have consistently been reported to be elevated in patients with type 2 diabetes mellitus in cross sectional studies [40,46,47], and have also been suggested to contribute to microangiopathy such as nephropathy in type 2 diabetes [48]. Moreover, in two prospective cohorts, elevated levels of IL-18 have been shown to predict the development of type 2 diabetes (Table 1) [49,50]. However, a major limitation of both studies is that fasting glucose or oral glucose tolerance tests were not performed at baseline evaluation: Elevated fasting glucose increases the risk of diabetes, and experimental hyperglycemia might increase circulating levels of IL-18 [51].

**Table 1:** Prospective studies evaluating the effect of elevated circulating IL-18 levels on cardiovascular, metabolic syndrome and diabetes related end points.

Study	Patients (n) and study population	Follow-up, years	End points	Outcome
Blankenberg et al. [56]	1229 (known CAD)	4	Cardiovascular mortality	Increased risk of cardiovascular mortality
Blankenberg et al. [58]	335 cases and 670 controls (healthy men)	5	CAD	Increased risk of CAD
Everett et al. [59]	253 cases and 253 controls (healthy women)	6	CVD	Increased risk of CVD
Koenig et al. [60]	382 cases and 1980 controls (population based)	11	CAD	No increased risk of CAD
Espinola-Klein et al. [62]	1263, stratified for MS (known CAD)	6	Cardiovascular mortality	Increased risk of cardiovascular mortality in MS strata
Trøseid et al. [63]	563, stratified for MS (elderly high risk men)	3	CVD	Increased risk of CVD in MS strata
Thorand et al. [50]	527 cases and 1698 controls (population based)	11	Type 2 diabetes	Increased risk of type 2 diabetes
Hivert et al. [49]	1012 cases and 1081 controls (women)	12	Type 2 diabetes	Increased risk of type 2 diabetes

CAD; coronary artery disease, CVD; cardiovascular disease, MS; metabolic syndrome.

### Interleukin-18 and cardiovascular disease

Studies regarding assocations between IL-18 and stable atherosclerosis have yielded conflicting results. One study showed that elevated levels of IL-18 were associated with the presence of subclinical atherosclerosis evaluated with intima media thickness of the carotid artery, also after adjustment for traditional risk factors, CRP and IL-6 [52]. On the other hand, in two large studies elevated levels of IL-18 were associated with carotid intima media thickness in univariate analyses, but not after adjustment for traditional risk factors [38,53]. Furthermore, in a study of patients with type 2 diabetes, both carotid intima media thickness and brachial-ankle pulse wave velocity were significantly associated with serum levels of IL-18, although not in multivariate analyses [54]. However, we recently showed that arterial stiffness measured by brachial pulse wave propagation was associated with IL-18 levels and components of the metabolic syndrome, even in multivariate analyses, both cross sectionally and during three years follow up [55].

Also data regarding IL-18 as a potential predictor of future cardiovascular events have so far been conflicting (Table 1). In patients with known coronary artery disease, circulating IL-18 levels as well as polymorphisms in the IL-18 gene were associated with future cardiovascular mortality [56,57]. In two prospective studies, elevated IL-18 levels were associated with future cardiovascular disease in previously healthy men [58] and women [59]. However, another large population based study with a follow up of 11 years showed that increased levels of IL-6 and CRP, but not IL-18 were associated with future coronary events [60]. Furthermore, a recent study from five European prospective CVD cohorts showed no association between polymorphisms in the IL-18 receptor genes and cardiovascular risk [61].

To date, only two studies have prospectively evaluated IL-18 as a potential predictor of cardiovascular events in populations with the metabolic syndrome (Table 1). In a large cohort consisting of men and women with known coronary artery disease, IL-18 was the only independent predictor of cardiovascular mortality in a subgroup with the metabolic syndrome, even after adjustment for CRP, IL-6 and fibrinogen [62]. In line with these results, we showed that IL-18 was a strong and independent predictor of cardiovascular events in elderly men with the metabolic syndrome, also after adjustment for CRP and IL-6, and with a synergistic effect of IL-18 and fasting glucose in the cardiovascular risk prediction [63].

### Potential mechanisms for interleukin-18 in the metabolic syndrome and atherosclerosis

#### Interleukin-18 in atherosclerotic lesions

IL-18 has been shown to be highly expressed in atherosclerotic plaques, mainly in plaque macrophages, and in particular in unstable plaques [64]. IL-18 is thought to exert its main pro-atherogenic effects by inducing IFN-γ production, which potentiates the inflammatory process and may lead to thinning or inhibition of the fibrous cap formation, resulting in vulnerable, rupture-prone plaques [65,66]. Furthermore, IL-18 seems to increase the expression of matrix metalloproteinases in vascular cells and macrophages, which might also contribute to plaque destabilisation [22,65].

Indeed, IL-18 might also directly cause plaque destabilisation and cardiac dysfunction. In a model with Apo E deficient mice, IL-18 overexpression enhanced the collagenolytic activity of smooth muscle cells, reduced intimal collagen content and fibrous cap thickness, leading to vulnerable plaque morphology [67]. Furthermore, in a mouse model with myocardial infarction, increased expression of cardiac IL-18 mRNA and a subsequent reduction of myocardial contractility were reported [68]. Interestingly, in a rat model with the metabolic syndrome, IL-18 overexpression aggravated insulin resistance, increased vascular inflammation and promoted remodeling by enhanced infiltration of macrophages and increased medial thickness in the aortic wall [69].

#### Interleukin-18 in adipose tissue

The classical perception of adipose tissue as a passive storage place of fatty acids has gradually been replaced by the notion of adipose tissue, and visceral fat in particular as an active endocrine organ. Visceral fat is now considered a central feature and potential cause of the metabolic syndrome [70], in part mediated by release of a large number of metabolically active substances known as adipokines. Adipokines are involved in several biological processes, including inflammation, thrombosis, insulin sensitivity and energy balance [71].

Human preadipocytes and adipocytes of all stages have been shown to spontaneously express and secrete IL-18 [23,72]. Of note, in obese individuals there is an increased expression of IL-18 in adipose tissue [36], and a 3-fold increased secretion from adipocytes compared with lean controls [23]. Interestingly, experimental hyperglycemia has been shown to increase the expression of IL-18 in adipocytes, an effect which was even more pronounced in the presence of intermittent hyperglycemia [73]. However, other studies have reported that non-adipocytes are the main sources of IL-18 in adipose tissue [24].

Whereas previous studies have reported macrophage infiltration in adipose tissue in obesity and insulin resistance [74,75], a recent study showed that infiltration of T-lymphocytes preceded the infiltration of macrophages in adipose tissue in early stages of insulin resistance in obese mice [76]. Notably, in human patients with type 2 diabetes from the same report, waist circumference correlated significantly with expression of IFN-γ in adipose tissue, suggesting a role of Th1 cells in insulin resistance [76]. Moreover, in another study of obese mice, there was a clear bias towards Th1 polarisation of lymphocytes in adipose tissue, which was associated with insulin resistance and could be reversed by immunotherapy [77]. IL-18 acts in synergy with IL-12 to stimulate Th1 polarisation [25], and levels of IL-12 have been reported to be increased in subjects with type 2 diabetes and by experimental hyperglycemia [78,79]. Hence, it could be speculated that IL-18 in combination with a hyperglycemic proinflammatory milieu might trigger Th1 activation and IFN-γ production [63], both in adipose tissue and in the atherosclerotic plaque.

#### Interleukin-18 in muscle tissue

Several reports have suggested that adipose tissue might not be the main source of IL-18 in patients with obesity and the metabolic syndrome [36,49,80,81]. In one study, plasma levels of IL-18 were reduced by weight loss, whereas no effect was seen on adipose tissue expression of IL-18 [36]. In another study, we showed that the reduction of IL-18 levels by exercise was significantly associated with improvement of the metabolic syndrome, but not with a reduction in visceral fat [81]. Hence, other body compartments might be involved.

There is increasing evidence that cytokines are involved in the regulation of skeletal muscle function, and tumor necrosis factor-α (TNF-α) has been associated with muscle catabolism and loss of muscle function [82]. IL-18 expression has been demonstrated in human skeletal muscles in a fiber specific way both in healthy individuals [83] and in patients with myopathy [67].

Recently, it was shown experimentally that TNF-α infusion induced reduced glucose uptake and increased IL-18 expression in human skeletal muscle tissue but not in adipose tissue, and the authors suggest that adipose tissue is unlikely to be a major source of IL-18 [80]. Still, it remains to demonstrate whether muscle tissue could be a major source of circulating IL-18 in subjects with and without the metabolic syndrome.

#### Interleukin-18 resistance

Paradoxically, genetically modified mice with IL-18 deficiency have been reported to develop hyperphagia, obesity and insulin resistance, which might be reversed by recombinant IL-18 administration [84]. Furthermore, it was shown that patients with obesity and type 2 diabetes produce significantly less IFN-γ in peripheral blood mononuclear cells in response to IL-18 stimulation compared to lean controls, most likely due to reduced expression of the IL-18 receptor β chain, and the authors have introduced a consept of IL-18 resistance as a potential explanation of elevated IL-18 levels in such patients [85]. This observation is supported by several studies that report defect leucocyte function and increased susceptibility to infections in patients with type 2 diabetes [86-88]. However, it remains to determine whether this immunological phenomenon translates into a similar resistance to the metabolic effects of IL-18 [85], and which organs that might be involved in such a process.

### Interleukin-18 as a potential therapeutic target

#### Effects of life style interventions

Lifestyle interventions consisting of diet and exercise have been shown to improve several cardiovascular risk factors including the metabolic syndrome and to reduce the risk of developing type 2 diabetes [89,90]. Hence, current guidelines for management of the metabolic syndrome highlight the combination of increased physical activity (at least 30 minutes on most days of the week) and improved diet (decreased intake of saturated fat and simple carbohydrates, increased intake of fruits, vegetables, whole grain and fish) to achieve a sustained weight loss and reversal of the components of the syndrome [2].

Weight loss mediated by calorie-restricted diet intervention was reported to decrease IL-18 levels in obese women [91]. Furthermore, combined interventions with diet and exercise have been shown to reduce IL-18 levels in both obese men [36] and women [92]. We have reported reduced serum levels of IL-18 by Mediterranean-like diet and omega-3 fatty acid supplementation in a popluation of elderly high-risk men [93]. In a post hoc analysis from the same trial, the reduction of IL-18 levels was associated with an increased number of metabolic syndrome components that improved during three years of intervention [55]. Moreover, Mediterranean-like diet was shown to reduce levels of CRP, IL-6 and IL-18 in a middle-aged population with the metabolic syndrome [94].

Aerobic exercise has been reported to reduce levels of CRP and IL-18 in subjects with type 2 diabetes [95,96]. In another study, aerobic exercise, but not strength training, reduced circulating levels of CRP, IL-6 and IL-18 in older subjects [97]. On the other hand, both endurance training and strength training reduced plasma levels of IL-18 in a cohort of HIV-infected patients with lipodystrophy [23,98]. Furthermore, exercise performed on rowing ergometer reduced adipose tissue expression of IL-18 in obese subjects [99]. Moreover, in a middle aged cohort of men with the metabolic syndrome, we have reported reduced levels of IL-18 associated with improvement of metabolic syndrome components by a combined intervention consisting of aerobic exercise and strength training [81].

#### Effects of drug therapy

Several drugs are relevant in the management of the metabolic syndrome, but this review will focus on the most commonly used first line drugs recommended in current guidelines [2], i.e. statins and Angiotensin Converting Enzyme (ACE)-inhibitors/Angiotensin II (ATII) receptor antagonists.

The effects of statin therapy on IL-18 levels have been conflicting. Some studies have shown reduced circulating levels of IL-18 in statin treated patients with hypercholesterolemia [100,101]. Furthermore, we showed a tendency to additive effect of exercise and pravastatin on serum levels of IL-18 in subjects with the metabolic syndrome [81]. On the other hand, one study showed no effect of 20 months treatment with atorvastatin in patients with stable coronary artery disease [102]. Moreover, statin therapy has consistently been reported to increase IL-18 in peripheral mononuclear cells [103-105]. Hence, the effect of statins on IL-18 levels remains elusive.

Although ACE-inhibitors and ATII receptor antagonists have been reported to have several anti-inflammatory properties, very few studies have evaluated the effect of these compounds on IL-18 levels. In experimental studies, ATII has been shown to increase IL-18 expression in vascular smooth muscle cells [106], whereas ATII receptor antagonists could inhibit aldosteron-induced IL-18 expression in cardiomyocytes [107]. However, candesartan treatment had no effect on circulating levels of IL-18 in patients with stable heart failure [108].

## Conclusions and future perspectives

Several lines of evidence support a pivotal role of IL-18 in the pathogenesis of the metabolic syndrome. Importantly, IL-18 has been shown to be closely associated with the metabolic syndrome and its components [14], to predict cardiovascular events and cardiovascular mortality in populations with the metabolic syndrome [62,63], and to precede the development of diabetes [49]. Still, the exact role of IL-18 in these conditions needs to be clarified.

Although life style interventions such as diet and exercise have been shown to reduce levels of IL-18 in populations with and without the metabolic syndrome, it remains to demonstrate that such a reduction translates into reduced incidence of diabetes and cardiovascular events. Furthermore, the contribution of adipose tissue, muscle tissue and other organs in regulating circulating levels of IL-18, as well as the potential role of IL-18 resistance require further investigation.

Since IL-18 is subject to several regulatory steps including cleavage by caspase-1, inactivation by IL-18 binding protein, and signalling via the β chain of the IL-18 receptor, it will be crucial to clarify to what extent circulating levels of total IL-18 relate to the biological actions of the cytokine. Finally, strategies for blocking IL-18 activity are currently investigated in various pathophysological conditions such as sepsis and heart failure [109], and could potentially represent future therapeutic tools for the metabolic syndrome and its consequences.

## Competing interests

The authors declare that they have no competing interests.

## Authors' contributions

MT drafted the manuscript. IS and HA critically reviewed the manuscript. All authors read and approved the final manuscript.
